# Crossing the Death Valley of publication process in cornea and ocular surface diseases: from abstracts presented at the Association for Research in Vision and Ophthalmology annual meeting to full-text manuscripts

**DOI:** 10.5935/0004-2749.20210019

**Published:** 2025-02-02

**Authors:** Giuseppe Giannaccare, Marco Pellegrini, Matilde Roda, Leonardo Taroni, Stefano Sebastiani, Federico Bernabei, Adriana Ferreira de Araujo Litvin, Fabiana Moscardelli, Emilio C. Campos

**Affiliations:** 1 Ophthalmology Unit, S.Orsola-Malpighi University Hospital, University of Bologna, Bologna, Italy; 2 Department of Ophthalmology, University Magna Graecia of Catanzaro, Italy; 3 Secretaria de Serviços Integrados de Saude Procudaria General de Republica, Italy

**Keywords:** Abstracting and indexing as topic, Bibliometrics, Congresses as topic, Meeting abstract, Publications/statistical & numerical data, Cornea, Resumos e indexação como assunto, Bibliometria, Congressos como assunto, Resumo de reunião, Publicações/estatística & dados numéricos, Córnea

## Abstract

**Purpose:**

This study was conducted to analyze the profile and publication rate of abstracts in indexed journals presented in the cornea section at the Association for Research in Vision and Ophthalmology Annual Meeting and to further identify potential predictive factors for better outcomes.

**Methods:**

Abstracts accepted for presentation at the 2013 Association for Research in Vision and Ophthalmology Annual Meeting in the cornea section were sought via PubMed and Scopus to identify whether they had been published as full-text manuscripts. First author’s name, time of publication, journal’s name, and impact factor were recorded. A multivariate regression was performed to explore the association between variables and both the likelihood of publication and the journal’s impact factor. A Kaplan-Meier analysis was performed to evaluate the time course of publication of abstracts.

**Results:**

Of the 939 analyzed abstracts, 360 (38.3%) were published in journals with a median impact factor of 3.4. The median time interval between abstract submission and article publication was 22 months. The multivariate analysis revealed that abstracts were more likely to be published if they were funded (OR=1.482, p=0.005), had a control group (OR=1.511, p=0.016), and had a basic science research scope (OR=1.388, p=0.020). The journal’s impact factor was higher in funded studies (β=0.163, p=0.002) but lower in multicenter studies (β=-0.170, p=0.001). The Kaplan-Meier analyses revealed significant differences in the publication time distribution for basic science vs clinical abstracts (χ^^2^^=7.636), controlled vs uncontrolled studies (χ^^2^^=6.921), and funded vs unfunded research (χ^^2^^=13.892) (p<0.05).

**Conclusion:**

Almost 40% of Association for Research in Vision and Ophthalmology abstracts were published within 5 years from submission. Funding support, basic research scope, and controlled design were the determinants of better outcomes of publication.

## Abstract (pt)

**Objetivo:**

Analisar o perfil e a taxa de publicação em periódicos indexados de resumos apresentados na seção de córnea da reunião anual da Association for Research in Vision and Ophthalmology - ARVO, para identificar potenciais fatores preditivos com objetivo de obter melhores resultados.

**Métodos:**

Artigos que foram aceitos para apresentação no encontro anual da Association for Research in Vision and Ophthalmology - ARVO 2013 na seção de córnea foram pesquisados via PubMed e Scopus para identificar se haviam sido publicados como manuscritos com texto integral. Nome do primeiro autor, data de publicação, nome da revista e fator de impacto foram registrados. Foi feita uma regressão multivariada para estabelecer uma associação entre as variáveis e a chance de publicação e o fator de impacto da revista. Foi utilizado o método Kaplan-Meier para analisar o tempo da apresentação até a publicação dos artigos.

**Resultados:**

Dos 939 artigos analisados, 360 (38.3%) foram publicados em revistas com um fator de impacto médio de 3.4. O intervalo de tempo entre a submissão do resumo e a publicação do artigo teve como mediana 22 meses. Na análise multivariada, resumos tinham mais chance de publicação se tinham algum tipo de financiamento (OR=1.482, p=0.005), tinham grupo controle (OR=1.511, p=0.016) e estavam no âmbito da pesquisa científica básica (OR+1.388, p=0.020). O fator de impacto da revista era maior em estudos financiados (β=0.163, p=0.002) e mais baixo naqueles multicêntricos (β=-0.170, p=0.001). A análise Kaplan-Meier mostrou diferenças significativas na distribuição de tempo até a publicação de resumos de ciência básica vs clínicos (χ^^2^^=7.636), com grupo controle vs sem grupo controle (χ^^2^^=6.921) e financiados vs não financiados (χ^^2^^=13.892) (p<0.05).

**Conclusão:**

Aproximadamente 40% dos resumos apresentados no encontro da Association for Research in Vision and Ophthalmology - ARVO foram publicados dentro de 5 anos da submissão. Financiamento, pesquisa no âmbito da ciência básica e presença de grupo controle foram fatores determinantes para melhores resultados em relação à chance de publicação.

## INTRODUCTION

Abstracts submitted to national and international meetings represent an important opportunity to present the current research activities and to gain useful feedback from the scientific community. The subsequent submission to a scientific journal through a peer-review process allows a more rigorous evaluation of the data presented, as well as their dissemination to a global audience through international search databases.

In general, the selection process of abstracts submitted to a meeting depends on a Scientific Committee that reviews and selects studies according to the potential scientific impact of the contribution. However, not all meeting abstracts end up being published in a peer-reviewed journal later. Previous studies have documented the publication rate of abstracts presented at national and international conferences of various specialties and have been used as an indicator of conference quality^(1-5)^. A systematic review indicated that the publication rate of accepted abstracts presented at overall biomedical congresses ranged from 22% to 63%, depending on the methodology, specialty, author experience, and results of the single research^(6)^. However, till date, very poor information is available regarding the publication out come of abstracts presented at international Ophthalmo logy Congresses^(7)^.

The aims of the present study were to investigate the (a) demographics of abstracts presented at the Association for Research in Vision and Ophthalmology (ARVO) 2013 Annual Meeting, (b) publication rate by peerreviewed journals, and (c) potential predictive factors for better outcomes of publication.

## METHODS

In this retrospective cohort study, abstracts presented at the ARVO 2013 Annual Meeting (Seattle, WA, USA) in the Cornea scientific section were identified and retrieved from the archive website. For each abstract, the following data were recorded into a database: abstract number, authors’ names, number of authors, affiliation of the first author, number of centers, sample size, main topic, research scope (basic science or clinical research), methodology (prospective or retrospective, randomized or nonrandomized, presence of a control group), and financial support.

A comprehensive literature search was then conducted using the PubMed-NCBI and Scopus databases. The search was restricted to the period between December 2012 (abstract submission deadline for ARVO 2013) and February 2018. Two independent investigators initially searched for the abstracts by combining the names of the first and last author. If no corresponding paper was found, the first author name was combined with various keywords from the title and the text of the abstract. The search was repeated for all possible keywords until either a match was found or all combinations were exhausted. Only publications with consistent hypotheses, study designs, and results were accepted as true matches. For each matching publication, the first author’s name, time of publication, journal’s name, and impact factor (IF) at the time of the publication were collected.

The SPSS statistical software (SPSS Inc, Chicago, IL) was used for data analysis. Univariate linear regression was performed to evaluate the association between variables and the likelihood of publication and between variables and the IF of the journal. A multivariate logistic regression and a stepwise linear regression were respectively performed to predict the publication rate to predict the IF of the journal, including independent variables that reached a significance level of <0.05 in the univariate analysis. A Kaplan-Meier analysis was performed to analyze the time course of the publication of abstracts. P<0.05 was considered to be statistically significant.

## RESULTS

**Figure 1:**
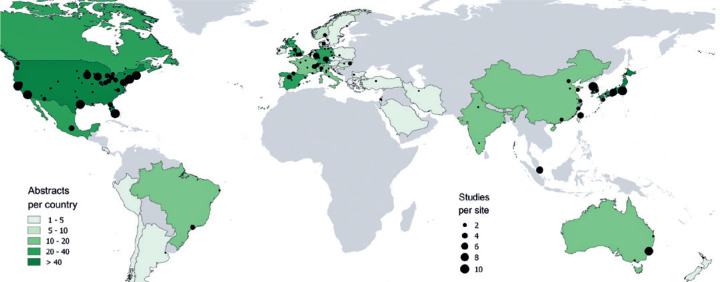
Map of abstracts and full-text articles. Map by country and study location of the abstracts and full-text articles. Country color refers to the number of abstracts per country, while circle size refers to the number of published studies for each site.

A total of 939 abstracts submitted to the ARVO 2013 Annual Meeting in the Cornea scientific section were included in this analysis. Table 1 shows the demographic characteristics of these abstracts. Regarding the geographic distribution of these abstracts, the affiliation of the first author spanned 41 different countries. In the majority of them, the affiliation was with an institution located in North America (531 abstracts; 56.6% of the total), followed by Europe (184 abstracts; 19.6%) and Asia (179 abstracts; 19.1%). South America, Oceania, and Africa were represented with 23 (2.5%), 22 (2.3%), and no abstracts, respectively (Figure 1).

**Table 1:** Demographics of abstracts accepted by ARVO 2013 in the Cornea scientific section

Characteristics	Number
Abstracts	939
Number of authors	5.0 ± 2.1
Number of centers	1.7 ± 1.1
Sample size	152.7 ± 653.7
Research scope Basic science	521
Clinical research	418
Design Prospective	800
Retrospective	139
Randomized	43
Nonrandomized	895
Controlled	174
Uncontrolled	765
Financial Support Yes	471
No	468

Of the analyzed abstracts, 360 (38.3% of the total) were published until February 2018. The number of authors during the publication process increased significantly from 5.0 ± 2.1 (mean ± standard deviation) in the abstracts to 6.2 ± 2.6 in the published articles (p<0.001). In 234 of the published articles (65%), the first author remained the same as that of the abstract, whereas in 126 studies (35%), there was a change in the first author. In particular, in 67 cases, the gender of the first author remained the same, whereas in 59 cases, there was a sex mismatch (36 cases from female to male, 23 cases from male to female). Journals with >1% of the total publications are listed in table 2. These 19 journals together accounted for 71.4% of all published articles. The median IF of the journals in which the abstracts were published was 3.4 (range 0-10.7). In total, 71.9% of the published abstracts appeared in journals related to the field of Ophthalmology. Among these, the official journals of the ARVO Society had the highest number of published articles (76; 21.1% of the total).

**Table 2:** Journals with the highest number of published articles (>1% of the total)

Journal name		Number		%
Investigative Ophthalmology and Visual Science		75	20.8	
PLOS ONE		24	6.7	
Cornea		24	6.7	
Eye and Contact Lens		18	5	
Experimental Eye Research		13	3.6	
Ophthalmology		13	3.6	
Journal of Cataract and Refractive Surgery		12	3.3	
American Journal of Ophthalmology		10	2.8	
Current Eye Research		10	2.8	
British Journal of Ophthalmology		8	2.2.	
Clinical Ophthalmology		8	2.2	
Contact Lens and Anterior Eye		6	1.7	
Ocular Surface		6	1.7	
Molecular Vision		6	1.7	
Optometry and Vision Science		6	1.7	
Scientific Reports		6	1.7	
Acta Ophthalmologica		4	1.1	
JAMA Ophthalmology		4	1.1	
Journal of Refractive Surgery		4	1.1	

Table 3 shows the number of abstracts grouped according to each main subsection along with the corresponding publication rate and the journal IF. There was no significant difference in the publication rate among the various main topics (χ^^2^^=18.487, p=0.071), whereas a statistically significant difference was found in the journal IF (p=0.003). In particular, the topic cell biology had the highest IF, whereas the topic contact lens had the lowest IF.

**Table 3:** Number of accepted abstracts, publication rate, and impact factor depending on the main topic

Main topic	No. of abstracts	Publication rate (%)	Impact factor	
Contact lens	118	31.4	2.4 ± 1.4	
Eye and lacrimal gland	203	35.5	3.2 ± 1.9	
Immunology, allergy, neovascularization	61	50.8	3.6 ± 1.5	
Keratoconus, cross-linking, and biomechanics	89	37.1	2.9 ± 1.4	
Corneal endothelium	58	44.8	3.1 ± 1.7	
Surgery: nonrefractive and keratoprosthesis	68	32.4	3.1 ± 2.3	
Cell biology	76	40.8	4.2 ± 1.5	
Corneal endothelium and imaging	56	53.6	3.6 ± 1.7	
Surgery: refractive	51	31.4	3.4 ± 1.8	
Corneal wound repair	71	39.4	3.4 ± 1.6	
Stroma, keratocytes, development, and dystrophies	53	32.1	3.8 ± 2.4	
Corneal surface in health and disease	35	48.6	3.5 ± 1.5	

A univariate binary logistic regression analysis was conducted to ascertain the effect of the variables on the likelihood that the abstracts were published (Table 4). Results showed that basic science studies had a significantly higher publication rate than clinical studies. Similarly, prospective, controlled, and financially supported studies had a higher publication rate. In contrast, multicenter studies had a lower publication rate than single-center studies. A multivariate logistic regression revealed that studies were more likely to get published if they were financially supported (OR=1.482, p=0.005), had a control group (OR=1.511, p=0.016), and had a basic science research scope (OR=1.388, p=0.020). The entire model was significant (p<0.001) and explained 33% of the variance in the publication rate.

**Table 4:** Univariate binary logistic regression of variables associated with abstract publication

Variable	Odds ratio (95% CI)	p-value
Number of authors	1.019 (0.958-1.084)	0.533
US affiliation	0.945 (0.726-1.230)	0.675
Sample size	1.000 (1.000-1.000)	0.226
Multicenter study	0.765 (0.586-0.999)	**0.049**
Basic science vs clinical research	1.497 (1.132-1.932)	**0.004**
Prospective vs retrospective	1.585 (1.072-2.344)	**0.021**
Randomized vs nonrandomized	0.857 (0.461-1.595)	0.627
Controlled vs noncontrolled	1.517 (1.088-2.115)	**0.014**
Financial support	1.614 (1.238-2.105)	**&lt;0.001**

**Figure 2:**
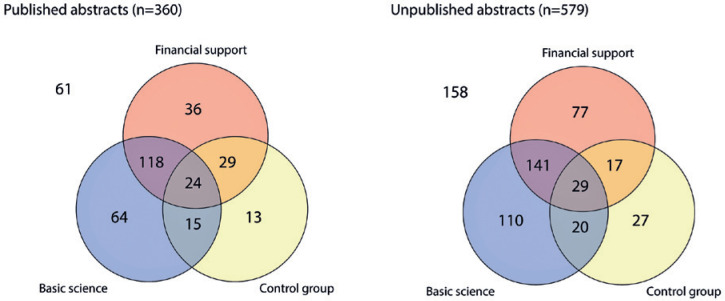
Venn diagram analysis. Venn diagram analysis of the number of abstracts with financial support, control group, and basic science research scope in the published and unpublished groups.

A Venn diagram analysis was performed to explore the effect of the financial support, the presence of a control group, and the basic research scope on the publication rate (Figure 2). The publication rate was 41.5% when at least one of the three parameters was present (i.e., the abstract was financially supported and had a basic research scope or a control group) and increased to 47.3% when all the three parameters were present.

**Figure 3:**
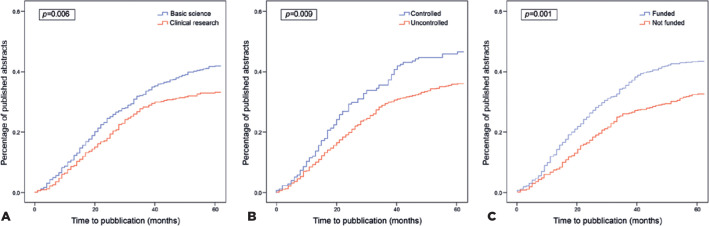
Kaplan-Meier analyses. A. Kaplan-Meier analyses of the publication process for basic science vs clinical abstracts; B. controlled vs uncontrolled abstracts; and C. funded vs unfunded abstracts.

The median time interval between abstract submission and article publication was 22 months (95% confidence interval: 20.0-24.1). Figure 3 shows the results of Kaplan-Meier analyses for the time course of the publication of abstracts based on the research scope (Part A), the presence of a control group (Part B), and the funding support (Part C). A log-rank test disclosed a statistically significant difference in the publication time distribution of basic science vs clinical abstracts (χ^^2^^=7.636, p=0.006), controlled vs uncontrolled abstracts (χ^^2^^=6.921, p=0.009), and funded vs not funded abstracts (χ^^2^^=13.892, p=0.001).

A univariate linear regression analysis performed to explore the association between abstract variables and journal IF (Table 5) revealed that studies with a higher number of authors and financial support were published in a journal with a higher IF, whereas multicenter studies were published in a lower IF journal. A multivariate stepwise linear regression demonstrated that the journal IF was higher in case of financially supported studies (β=0.163, p=0.002) and lower for multicenter studies (β=-0.170, p=0.001). The entire model was significant (p<0.001) and explained 6% of the variance in the IF.

**Table 5:** Univariate linear regression of variables associated with impact factor

Variable	β (95% CI)	p-value
Number of authors	0.107 (0.002-0.171)	**0.044**
US affiliation	-0.016 (-0.366-0.360)	0.987
Sample size	-0.043 (0.000-0.000)	0.597
Multicenter study	-0.184 (-1.003- -0.286)	**<0.001**
Basic science vs clinical research	0.009 (-0.342-0.404)	0.869
Prospective vs retrospective	0.011 (-0.517-0.636)	0.840
Randomized vs nonrandomized	0.032 (-0.577-1.085)	0.548
Controlled vs noncontrolled	0.071 (-0.137-0.729)	0.179
Financial support	0.178 (0.265-0.988)	**0.001**

## DISCUSSION

Dissemination of the data obtained from a research project generally begins with the presentation of an abstract at a meeting and often culminates with the subsequent complete publication in a peer-reviewed journal. Previous studies analyzing the publication outcomes of meeting abstracts had identified different characteristics of the abstracts that were associated with higher publication rates^(1-5)^. A recent investigation on the abstracts presented at the American Academy of Ophthalmology (AAO) 2008 Annual Meeting reported a publication rate of 39.1% and demonstrated that the factors that correlated with a higher publication rate were oral presentations focused on certain types of subspecialty^(7)^. Furthermore, a focus on rare diseases, affiliation with a US-based institute, and funding correlated with publication in journals with a higher IF.

In the present study, we analyzed the publication outcomes of approximately 1000 abstracts presented at the Cornea scientific section of the ARVO 2013 Annual Meeting. Because almost half of the randomized clinical trials presented at the ARVO 2001-2004 Annual Meetings have been shown to exhibit some degree of discordance compared with subsequent publications^(8)^, we considered only full-text manuscripts with consistent hypotheses, designs, and results as true matches. We determined an overall publication rate of 38.3%, which was similar to that reported for the AAO 2008 Annual Meeting^(7)^ but slightly lower than that reported from a Cochrane systematic review incorporating almost 30,000 abstracts from overall biomedical meetings^(6)^.

More than two-thirds of the published articles were disseminated in journals related to the ophthalmic field, with the highest number being published in the official journals of the ARVO Society. The median IF of the overall journals in which the abstracts were published was 3.4. This value is slightly higher than the median IF re ported for papers originated from AAO abstracts (2.9)^(7)^. This result is even more significant considering that the number of ARVO abstracts presented in the Cornea section is similar to the total number of the overall AAO abstracts, irrespective of the topic. Although the IF is an indicator that suffers from some limitations, it is valued quite strongly with regard to the perceived quality of the journal^(9)^. A relatively high IF of the published ARVO abstracts may depend on the fact that some articles were published in basic science journals with a very high IF, even when the majority of them were published in ophthalmological journals.

Previous studies investigating the relationship between the geographic distribution of abstracts and their likelihood of publication have reported contradictory conclusions^(7,10,11)^. In our analysis, the first author in more than half of the abstracts was affiliated with an institution located in North America. However, there was no association between the nationality of the first author and the publication rate or the journal IF.

An evaluation of the potential predictors of publication may provide valuable information for scientists approaching toward the preparation and submission of abstracts. In the present study, the parameters of financial support, presence of a control group, and basic research scope were associated with a higher likelihood of the abstracts being published. In particular, the publication rate increased to 47.3% for the abstracts that were financially supported and had a control group and a basic research scope.

Financially supported abstracts were also published in journals with a higher IF than non-supported studies, which is consistent with the results reported by Mimouni et al.^(7)^. This finding may be the result of the abundant resources provided to the researchers in case of funded studies. Moreover, the funding party may operate a filtering process, thereby granting financial support only to the studies considered as worth investigating. The financial sponsorship of clinical trials provided by pharmaceutical industries has increased significantly over the years^(12)^. Despite an increasing focus on the transparency surrounding financial conflict of interest, the role of pharmaceutical industries in the production of scientific evidence still represents a relevant concern for several researchers. Previous studies have demonstrated that trials funded by pharmaceutical industries are more likely to be associated with statistically significant proindustry findings^(13-15)^. The possible explanations include publication bias and the selection of an inappropriate comparator to the product being investigated.

Abstracts that had a basic (as opposed to clinical) research scope were more likely to get published. This finding is consistent with the results of a previous systematic review that analyzed almost 15,000 abstracts submitted to dozens of meetings^(16)^. The authors of that review hypothesized that basic and clinical research studies may differ in terms of both quality of conduction of the study and presentation of the results. However, the existence of a bias in favor of basic research cannot be excluded, as previous studies have emphasized the tendency of chairpersons and senior research advisors to perceive basic research activities of higher quality compared with clinical research activities^(17)^.

In general, a rigorous method for scoring the quality of a research is to assign an appropriate level of evidence according to the study design. In the present study, abstracts that had a control group exhibited a higher publication rate than uncontrolled studies. This result was not surprising, because in the hierarchical system of classifying evidence, controlled studies are deemed to have higher quality than uncontrolled studies^(18)^.

The data derived from this study might help young researchers when approaching toward the conception of a research project and later to the preparation and submission of an abstract. However, they should not forget that the ultimate aim of a biomedical research is not to generate publications anyhow but to add novel data that could be useful to improve the current understanding, diagnosis, and treatment of diseases.

The major limitation of this study is the lack of information concerning the reasons for unpublished abstracts. In fact, it would be interesting to analyze the number of abstracts that were not submitted for publication at all or those that did not survive the peer-review process. A previous study surveyed those authors whose abstracts were presented at an orthopedic meeting and never published as full publications. Only few authors had confirmed that their manuscripts were submitted and rejected, whereas more than one-third of them stated that they never submitted the abstract for full publication^(19)^. The common barriers to publication were the lack of time and interest for full publication and the difficulties in collaboration among co-authors. Another limitation of this study is associated with the fact that the academic title of the first author of the abstracts was not available, which consequently hampered the inclusion of this additional parameter in the predictive analysis.

Almost 40% of the abstracts accepted in the scientific section of Cornea of the ARVO 2013 annual meeting were published in peer-reviewed journals within 5 years from submission at the congress. The parameters associated with a higher publication rate were funding support, basic research scope, and the presence of a control group.
